# The Physical Activity and Fitness in Childhood Cancer Survivors (PACCS) Study: Protocol for an International Mixed Methods Study

**DOI:** 10.2196/35838

**Published:** 2022-03-08

**Authors:** Hanne C Lie, Sigmund Anderssen, Corina Silvia Rueegg, Truls Raastad, May Grydeland, Lene Thorsen, Trine Stensrud, Elisabeth Edvardsen, Marie Hamilton Larsen, Ingrid Kristin Torsvik, Lars Peder Bovim, Miriam Götte, Päivi Maria Lähteenmäki, Susi Kriemler, Hanne Bækgaard Larsen, Martin Kaj Fridh, Kristin Ørstavik, Henrik Brun, Iren Matthews, Else Hornset, Ellen Ruud

**Affiliations:** 1 Department of Behavioural Medicine, Institute of Basic Medical Sciences, Faculty of Medicine University of Oslo Oslo Norway; 2 Department of Sports Medicine Norwegian School of Sport Sciences Oslo Norway; 3 Oslo Centre for Biostatistics and Epidemiology Oslo University Hospital Oslo Norway; 4 Department of Physical Performance Norwegian School of Sport Sciences Oslo Norway; 5 National Advisory Unit on Late Effects after Cancer Treatment, Department of Oncology Division of Cancer Medicine Oslo University Hospital Oslo Norway; 6 Department of Clinical Service Division of Cancer Medicine Oslo University Hospital Oslo Norway; 7 Department of Pediatrics Haukeland University Hospital Bergen Norway; 8 Department of Health and Functioning Faculty of Health and Social Sciences Western Norway University of Applied Sciences Bergen Norway; 9 Department of Pediatric Hematology/Oncology Clinic for Pediatrics III, West German Cancer Center University Hospital Essen Essen Germany; 10 Department of Pediatric and Adolescent Hematology/Oncology Turku University Hospital University of Turku Turku Finland; 11 Epidemiology, Biostatistics and Prevention Institute University of Zurich Zurich Switzerland; 12 Department of Pediatrics and Adolescent Medicine Copenhagen University Hospital (Rigshospitalet) Copenhagen Denmark; 13 Institute for Clinical Medicine Faculty of Health Science The University of Copenhagen Copenhagen Denmark; 14 Department of Neurology Section for Clinical Neurophysiology Oslo University Hospital Oslo Norway; 15 Department of Pediatric Cardiology Oslo University Hospital Oslo Norway; 16 Department of Paediatric Allergy and Pulmonology Oslo University Hospital Oslo Norway; 17 Norwegian Childhood Cancer Society Oslo Norway; 18 Department of Pediatric Hematology and Oncology Oslo University Hospital Oslo Norway; 19 Institute for Clinical Medicine Faculty of Medicine University of Oslo Oslo Norway

**Keywords:** childhood cancer survivor, physical activity, physical fitness, barriers, intervention, quality of life, fatigue

## Abstract

**Background:**

Survivors of childhood cancer represent a growing population with a long life expectancy but high risks of treatment-induced morbidity and premature mortality. Regular physical activity (PA) may improve their long-term health; however, high-quality empirical knowledge is sparse.

**Objective:**

The Physical Activity and Fitness in Childhood Cancer Survivors (PACCS) study comprises 4 work packages (WPs) aiming for the objective determination of PA and self-reported health behavior, fatigue, and quality of life (WP 1); physical fitness determination (WP 2); the evaluation of barriers to and facilitators of PA (WP 1 and 3); and the feasibility testing of an intervention to increase PA and physical fitness (WP 4).

**Methods:**

The PACCS study will use a mixed methods design, combining patient-reported outcome measures and objective clinical and physiological assessments with qualitative data gathering methods. A total of 500 survivors of childhood cancer aged 9 to 18 years with ≥1 year after treatment completion will be recruited in follow-up care clinics in Norway, Denmark, Finland, Germany, and Switzerland. All participants will participate in WP 1, of which approximately 150, 40, and 30 will be recruited to WP 2, WP3, and WP 4, respectively. The reference material for WP 1 is available from existing studies, whereas WP 2 will recruit healthy controls. PA levels will be measured using ActiGraph accelerometers and self-reports. Validated questionnaires will be used to assess health behaviors, fatigue, and quality of life. Physical fitness will be measured by a cardiopulmonary exercise test, isometric muscle strength tests, and muscle power and endurance tests. Limiting factors will be identified via neurological, pulmonary, and cardiac evaluations and the assessment of body composition and muscle size. Semistructured, qualitative interviews, analyzed using systematic text condensation, will identify the perceived barriers to and facilitators of PA for survivors of childhood cancer. In WP 4, we will evaluate the feasibility of a 6-month personalized PA intervention with the involvement of local structures.

**Results:**

Ethical approvals have been secured at all participating sites (Norwegian Regional Committee for Medical Research Ethics [2016/953 and 2018/739]; the Oslo University Hospital Data Protection Officer; equivalent institutions in Finland, Denmark [file H-19032270], Germany, and Switzerland [Ethics Committee of Northwestern and Central Switzerland, project ID: 2019-00410]). Data collection for WP 1 to 3 is complete. This will be completed by July 2022 for WP 4. Several publications are already in preparation, and 2 have been published.

**Conclusions:**

The PACCS study will generate high-quality knowledge that will contribute to the development of an evidence-based PA intervention for young survivors of childhood cancer to improve their long-term care and health. We will identify physiological, psychological, and social barriers to PA that can be targeted in interventions with immediate benefits for young survivors of childhood cancer in need of rehabilitation.

**International Registered Report Identifier (IRRID):**

DERR1-10.2196/35838

## Introduction

### Background

Improved diagnostics and treatment of pediatric cancer over the past decades have drastically increased 5-year survival rates to >80% [1]. Therefore, the population of survivors is rapidly growing, and there are currently an estimated ≥500,000 survivors of childhood cancer in Europe [2]. Survivors of childhood cancer represent a vulnerable population, which is expected to face high, long-term risks of morbidity [3,4] and early mortality because of the late effects of cancer and its treatment [5-7]. Late effects can arise years or decades beyond treatment completion; by the age of 50 years, approximately all survivors of childhood cancer experience multiple chronic health conditions [8]. Late effects include, among others, cardiorespiratory disorders, endocrine dysfunction, secondary cancers, musculoskeletal deficits, metabolic syndrome, early frailty, neurocognitive impairments, and fatigue, resulting in reduced quality of life (QoL) [3,4,8]. Increased knowledge of the negative consequences of treatment has prompted efforts to improve the long-term QoL and health outcomes of survivors of childhood cancer.

There is increasing evidence for a range of positive effects of regular physical activity (PA) on reducing risks of late effects, early mortality, and improving QoL in both adult [9-16] and young survivors of childhood cancer [10,16,17]. However, reduced levels of PA and physical fitness (PF) in survivors of childhood cancer compared with controls have been consistently reported, albeit with substantial heterogeneity across studies [9]. Objectively measured PA levels in childhood and early adolescent survivors of childhood cancer support these findings, suggesting considerably reduced PA levels compared with controls in the small-scale studies that exist [18]. Moreover, studies among adult survivors of childhood cancer suggest that reduced PA levels persist for a substantial proportion of survivors well into adulthood [19,20]. Thus, there is a need for effective PA interventions among young survivors of childhood cancer to establish healthy PA routines at an early age. A modest body of research indicates the effectiveness of PA interventions on PA behavior and physical, psychosocial, cognitive, and social outcomes for groups of young survivors of childhood cancer [10,16,17]; however, PA recommendations are currently limited by important knowledge gaps and methodological shortcomings, including low sample sizes, single-center samples, and large heterogeneity across studies [9,10,16,17]. Data from large-scale studies of survivors of childhood cancer representing multiple diagnostic groups and countries, using device-based measurements, are still lacking.

Knowledge gaps regarding PF in young survivors of childhood cancer are even more prevalent than for PA [21]; however, findings indicate that PF is also persistently poorer among young survivors of childhood cancer with reduced cardiorespiratory fitness (9%-23%) [22,23] and muscle strength (10%-20%) [9,24] than the age-matched controls. Interestingly, the difference in PF between survivors of childhood cancer and healthy controls seems to progress years after treatment [22]. Research within our group suggests that Norwegian adult survivors of childhood leukemia and lymphoma experience reduced exercise capacity because of the cardiotoxic effect of treatment [25] and lower QoL compared with cancer-free controls even >20 years after diagnosis [26]. Thus, low levels of PA and PF may exacerbate already increased risk of late effects, functional limitations, and reduced QoL for survivors of childhood cancer well into adulthood [27,28].

Research on young survivors’ perspectives on what facilitates and hinders participation in PA is also scarce [29,30]. The observed lower levels of PA among young survivors of childhood cancer than in the general population are often attributed to a range of physical and psychosocial factors, including (1) adverse consequences of cancer therapies on PF and function [17]; (2) anxiety and overprotective attitudes toward PA [31]; and (3) the cancer and treatment may coincide with the period in life when children start organized sports, thus contributing to an ability gap to healthy peers [22]. Late effects and PF deficits interfere with physical, psychological, and social functioning and the ability to participate in daily and social activities in approximately 50% of adult survivors of childhood cancer [32]. Therefore, limitations to engaging in PA can create a *vicious circle* of reduced activity levels, which further negatively influence long-term development, health, and functioning. Therefore, knowledge of perceived barriers and facilitators for survivors of childhood cancer regarding PA and exercise programs is needed to develop effective and sustainable PA interventions.

### Objectives

The overall aim of the Physical Activity and Fitness in Childhood Cancer Survivors (PACCS) study is to address the abovementioned research gaps and generate knowledge that will contribute to the development of an evidence-based PA intervention for young survivors of childhood cancer. The PACCS study is run by an international multidisciplinary consortium, including Norway, Finland, Denmark, Germany, and Switzerland, and includes four work packages (WPs) with the following main objectives:

1. Objectively determine levels of PA and sedentary time among survivors of childhood cancer compared with reference data and identify physiological, psychological, and social correlates, including QoL and fatigue (WP 1)
2. Objectively determine PF and identify physical and physiological factors limiting PF in survivors of childhood cancer compared with controls (WP 2)
3. Identify physical, psychological, social, and contextual factors that act as barriers to and facilitators for engagement in PA in survivors of childhood cancer; furthermore, to identify opportunities for collaboration between specialist and municipality health care services to establish systematic PA interventions locally for survivors of childhood cancer (WP 3)
4. Develop and feasibility test a PA intervention as a preparation for a future well-designed large randomized controlled trial based on results from WP 1 to 3 (WP 4)

## Methods

### Study Design, Study Population, and Inclusion Criteria

**Figure 1:**
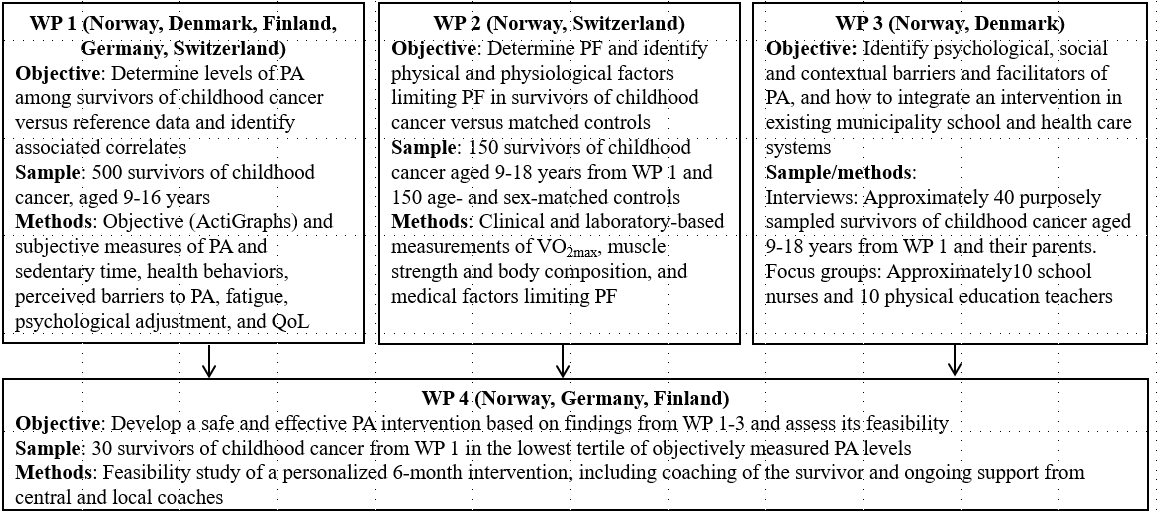
Overview of the objectives and methods for the 4 work packages (WPs) of the Physical Activity and Fitness in Childhood Cancer Survivors study.

The PACCS study [33] is an international multicenter study based on a mixed methods design that combines clinical and physiological assessment methods, patient-reported outcome measures, qualitative interviews, and a feasibility intervention. WPs 1, 2, and 3 have a cross-sectional design, whereas WP 4 will be a single-arm (intervention only), multicenter, open PA feasibility trial. The study population comprises young survivors of childhood cancer (aged 9-18 years) recruited from seven medical centers in Europe: 2 in Norway (Oslo and Bergen) and Finland (Turku and Tampere) and 1 in Denmark (Copenhagen), Germany (Essen), and Switzerland (Basel). Survivors of childhood cancer with *any* malignant disease, at least 1 year after completion of cancer treatment, and those attending outpatient follow-up care consultations are eligible for inclusion. Survivors of childhood cancer will be excluded if there are language difficulties or limited cognitive functioning. Figure 1 provides an overview of the different WPs.

We aimed to include a minimum of 500 survivors of childhood cancer aged 9 to 16 years in WP 1. We aimed to consecutively enroll approximately 150 participants from WP 1 to WP 2. The age group in WP 2 will be 9 to 18 years, as 1 to 2 years may, in some sites, elapse from inclusion into WP 1 to participation in WP 2. In addition, in WP 2, 150 age- and sex-matched controls will be enrolled. A subsample of 40 selected WP 1 participants and one of their parents will be invited to participate in qualitative interviews in WP 3. Participants will be purposely sampled to maximize the variation of key sociodemographic and clinical factors, including activity levels and rural or urban places of residence. Approximately 10 school nurses and 10 physical education teachers will also be recruited. In WP 4, around 30 survivors of childhood cancer aged 12 to 18 years within the lowest tertial of total PA, as assessed by the accelerometer in WP 1, without contraindications for vigorous PA, will be recruited among the participants of WP 1. As WP 4 is a pilot feasibility study, we aim to recruit survivors of different diagnoses, age ranges, and genders. All countries and study sites will participate in WP 1; Norway and Switzerland will participate in WP 2; Norway and Denmark will participate in WP 3; and Norway, Finland, and Germany will participate in WP 4.

### Assessments, Power Calculations, and Statistical Analyses

#### WP 1

##### Overview

Outcomes of WP 1 include objective and subjective assessments of PA and sedentary behavior, barriers to PA, fatigue, psychological adjustment, and QoL. The self-reported measures, as well as demographic variables, will be collected using electronic questionnaires. An overview of the key variables assessed in WP 1 is presented in Table 1.

**Table 1:** Overview of factors assessed at each study site for WPs^a^ 1, 2, and 4.

Variables			WP 1; all sites		WP 2							WP 4; Oslo, Bergen, Turku, and Essen
					Oslo		Bergen		Basel			
**Objectively assessed**												
	Physical activity (counts per minute; sedentary time; light, moderate, and vigorous activity)^b^	✓		✓		✓		✓		✓		
	Echocardiography			✓		✓						
	Lung function			✓		✓		✓				
	Neurological tests			✓		✓						
	Cardiopulmonary Exercise Testing			✓^c^		✓^c^		✓^d^		✓^c^		
	Knee extension strength			✓		✓		✓		✓^e^		
	Hand grip			✓		✓		✓				
	Chest press strength			✓		✓		✓		✓^f^		
	Countermovement jump			✓		✓		✓				
	1-Minute sit-to-stand test			✓		✓		✓		✓		
	Body composition (DXA^g^)			✓		✓		✓				
	Muscle thickness (ultrasound)			✓		✓						
	Blood volume and hemoglobin mass			✓								
	Anthropometric variables (height and weight)	✓		✓		✓		✓		✓		
	Puberty stage (Tanner)			✓		✓		✓				
	Medical variables (diagnosis, time since diagnosis, treatment, time since treatment, medication use, and comorbidities)	✓		✓		✓		✓				
**Questionnaires**												
	Demographic variables (age and gender)^h,i^	✓		✓		✓		✓		✓		
	Self-reported PA^j,k^, including recreational sports participation and active commuting habits^h,i^	✓										
	Physical education^l^	✓										
	Perceived PA barriers: 15 items covering reasons for not being physical active^m^	✓								✓		
	Attitude toward PA (perceived physical competence, perceived enjoyment, and motivation)									✓		
	Television or screen time^n^	✓										
	Diet, including sugar rich beverages^h,o^	✓										
	Sleep^i^	✓										
	Quality of life (PedsQL^p^)	✓								✓		
	Fatigue (PedsQL MDF^q^ scale)	✓								✓		
	Psychological adjustment (SDQ^r^)	✓										

^a^WP: work package.

^b^For WP 1, 2, and 4: ActiGraph model GT3X+, ActiGraph LLC; for WP 4: real-time tracking of PA over 6 months (time spent in activity, type of activity, and intensity and steps) using a Polar watch (Polar Global).

^c^Cardiopulmonary exercise testing on a treadmill.

^d^Cardiopulmonary exercise testing on a bike.

^e^Isometric knee extension strength is tested on the custom-built strength ergometer similar to WP 2 in Bergen and Oslo and on sites' own commercial devices in Turku and Essen.

^f^Upper arm strength is tested as isometric chest press on the custom-built strength ergometer similar to WP 2 in Bergen and Oslo and on sites' own commercial device in Essen. Turku performed a dynamic upper extremity lifting test in a standing position.

^g^DXA: dual-energy x-ray absorptiometry.

^h^Items from the Health in Adolescents study [34].

^i^Items from the Physical Activity in Norwegian Children Study [35].

^j^PA: physical activity.

^k^Number of counts per minute (cpm; primary outcome); fulfillment of physical activity (PA) recommendations (>60 minutes of moderate to vigorous PA per day); PA intensity categories (counts translated into metabolic energy equivalents of intensities): sedentary time (<100 cpm), light (100-1999 cpm), moderate (2000-5999 cpm), and vigorous (≥6000 cpm).

^l^Items from the Relevance of Physical Activity Contexts in the Everyday Life of Adolescents study [36].

^m^Constructed for the purpose of the Physical Activity and Fitness in Childhood Cancer Survivors study based on literature review and expert and user input.

^n^Items from the Health Behavior in School-aged Children study [37].

^o^Items from the UngKost [38].

^p^PedsQL: Pediatric Quality of Life Inventory [39].

^q^PedsQL MDF: Pediatric Quality of Life Inventory Multidimensional Fatigue scale [40].

^r^SDQ: Strength and Difficulties Questionnaire [41].

##### Assessment of PA and Sedentary Time by Accelerometer

PA and sedentary time will be assessed objectively by accelerometers worn for 7 days during the awake time to measure accelerations of +6 G to –6 G (ActiGraph model GT3X+, ActiGraph LLC). The sample rate of the accelerometer is set to measure the raw signals at 30 Hz. Total PA will be described as the number of counts per minute (cpm), which is a measure of the volume of PA assessed by the accelerometer. The accelerometer data will be uploaded by all collaborators to a central server located at the Norwegian School of Sport Sciences and analyzed at one site. Accelerometer measurements with at least four valid measurement days (≥480 min/day) will be included in the analysis.

The activity counts from the accelerometers will be translated into metabolic energy equivalents of intensities as sedentary time (<100 cpm), light (100-1999 cpm), moderate (2000-5999 cpm), and vigorous (>6000 cpm) PA [42]. Participants will be categorized as reaching the international recommendations for PA if they engage in at least 60 minutes of moderate to vigorous PA (≥2000 cpm) per day on average over valid accelerometer measurement days.

##### Questionnaires

###### Overview

All questionnaires will be completed electronically using tablet computers. Different questionnaires are available for survivors of childhood cancer aged 9 to 12 years and their parents (proxy reports) and survivors of childhood cancer aged 13 to 16 years and their parents. The questionnaires comprise mainly validated and standardized questions (Table 1). Questionnaires will be translated into local language if validated translations do not exist, following recommended forward–backward translation procedures. Questionnaire data will be directly uploaded and stored on the secured server for sensitive data at the University of Oslo’s Center for Information Technology.

###### Self-reported PA and Exercise

Items pertaining to exercise in leisure time will be measured by questions extracted from studies of age- and sex-stratified reference material (the Health in Adolescents study, Physical Activity among Norwegian Children study wave 2 [35], and the Relevance of Physical Activity Contexts in the Everyday Life of Adolescents [REPAC]; the REPAC study described below [34-36]) to enable comparisons. The questions target PA and include frequency, duration, and type of PA or exercise and active transportation (questionnaires are available upon request).

###### Physical Education in School

Questions regarding attitudes and participation toward physical education before and after the cancer experience are based on the questionnaire of an ongoing study of approximately 3000 Norwegian adolescents (REPAC study) [36].

###### Perceived PA Barriers

Perceived barriers to being physically active will be assessed using a self-constructed questionnaire. Survivors of childhood cancer will rate degree of agreement with 15 statements in response to the question “I am less physically active than I want to because;....” The 15 statements reflect potential physical, psychosocial, and mental barriers to PA and were designed based on clinical experience, input from user representatives, and pilot testing. Examples of statements are “I don’t have time for it; I don’t know how active I can or should be; My body feels too heavy; I am too fatigued; I have pain; My parents tell me to be careful.” Each statement is scored on a 5-point Likert-type scale ranging from *totally disagree* to *totally agree*.

###### Screen Time

Screen time (time spent in front of a computer, watching television, playing computer games, surfing on the internet, and chatting) will be assessed by 2 questions from a questionnaire used in a large international study on children and adolescents (the Health Behavior in School-aged Children study) [37].

###### Diet and Nutrition

A total of 3 questions regarding diet were extracted from a nationally representative study on children and adolescents’ dietary habits, called the Norwegian Ungkost study [38].

###### Sleep

Participants will report when they usually get out of bed and when they go to bed on school days [43].

###### QoL Questionnaire

QoL will be measured using the Pediatric QoL Inventory (PedsQL) core module [39]. The PedsQL includes four subscales—physical, emotional, social, and school functioning—and has 2 different modules validated for children aged 8 to 12 years and adolescents aged 13 to 18 years. The PedsQL has been used in children with cancer [40]. In addition, parents will be asked to complete the parent module (proxy report) of the PedsQL.

###### Fatigue

Fatigue will be measured using the PedsQL Multidimensional Fatigue Scale, which measures general fatigue, sleep/rest fatigue, and cognitive fatigue [40]. Parents will complete the respective parent modules.

###### Psychological Adjustment

The Strength and Difficulties Questionnaire (SDQ) will be used to measure psychological adjustment and emotional problems. The SDQ is widely used in children aged 8 to 17 years [41].

###### Other Variables

Parents will provide sociodemographic variables in their questionnaire (child’s age, child’s sex, parents’ education, and parents’ income).

##### Information Extracted From Medical Records

The following variables will be collected from medical records and stored on Services for Sensitive Data (TSD): sex, age at diagnosis, cancer diagnosis, month and year of diagnosis, month and year when completed treatment, type of treatment (chemotherapy, surgery, radiation [dose—in Gy—and area], and stem cell transplantation), recurrence, diagnosis and treatment before or after puberty, weight, and height at the time of the study.

##### Reference Material

Age- and sex-stratified reference material for the primary and most of the secondary outcomes are available for comparison of marginal means and will be provided from several large-scale studies among healthy children and adolescents. References on PA and sedentary time will be available from two Norwegian studies—the Physical Activity among Norwegian Children study wave 2 (N=3538; age 9 and 15 years) [35], Health in Adolescents study (N=1528; age 11 and 13 years) [34]—and one German study—the German Health study (N=1500-2000; age 6 to 17 years) [44]. Age- and sex-stratified national reference materials are also available for attitudes toward physical education (REPAC) [36], screen time (Health Behavior in School-aged Children study) [37], and psychosocial measures (PedsQL [45], PedsQL Multidimensional Fatigue Scale [46], and SDQ [47]).

##### Power Calculation and Statistical Analyses WP 1

From studies in the general Norwegian population, we know that children aged 9 to 15 years engage in an average of 549 (SD 180) cpm (measured by accelerometer) [35]. We assumed reduced PA levels in survivors of childhood cancer. To estimate a difference of 10% in objectively measured PA between survivors of childhood cancer and healthy controls (494 vs 549 cpm, SD 180 for both; power=80%; significance level *P*<.05, one-sided), we need to include 250 survivors of childhood cancer. Given the heterogeneity in the expected sample (age and diagnoses) and to facilitate subgroup comparisons, we aim to include at least n=500 in WP 1 to facilitate subgroup comparisons.

Descriptive statistics will be used to present the levels of PA (including different intensity measures and activity patterns) and sedentary time of survivors of childhood cancer compared with the reference materials and PA recommendations. Multilevel models (mixed models) will be used to adjust for the different study sites as random intercepts. We will perform univariable and multivariable multilevel regression models (linear, logistic, or ordinal depending on the outcome) to (1) assess the clinical and sociodemographic factors associated with meeting the PA recommendations in survivors of childhood cancer; (2) assess clinical and sociodemographic factors associated with different patterns or levels of PA and sedentary time; and (3) assess the associations among PA patterns or levels (exposure) and QoL, fatigue, and other health behaviors.

#### WP 2

##### Overview

###### Measures

Outcomes of WP 2 include measurements of cardiorespiratory fitness, muscle strength, power, and muscular endurance. Norway (Oslo and Bergen) and Switzerland (Basel) will participate in WP 2 (Table 1 provides an overview of tests and self-reported measures performed at each site specific to WP 2). In Norway, age- and sex-matched controls will be the same-age friend or sibling of the survivor of childhood cancer, chosen and invited by the survivors of childhood cancer themselves. In Switzerland, age- and sex-matched controls will be recruited from siblings or friends of survivors or within the network of the Swiss PACCS study team.

###### Cardiorespiratory Fitness

Before testing, survivors of childhood cancer will undergo a cardiological screening. Peak oxygen uptake (VO_2_peak) will be assessed using a cardiopulmonary exercise test (CPET). In Norway, the CPET will be performed by walking and running on a treadmill using a child-friendly continuous graded treadmill protocol until exhaustion [48], whereas in Switzerland, the CPET will be performed on a cycle ergometer using an incremental bike protocol from Godfrey [49]. The starting workload after habituation to the treadmill will be 3.0 km/hour at 0% inclination. The workload will then be increased every minute by alternately increasing the speed by 1 km/hour and the inclination by 2% every other minute. For the bike protocol, the initial workload will be set to 10, 15, or 20 W depending on the child’s height and increased by 10, 15, or 20 W every minute, respectively. The participants will be encouraged to keep a pedaling frequency of 60 to 90 revolutions per minute. Gas exchange and ventilatory variables will be measured continuously, breath by breath, using a Hans Rudolph 2-way breathing mask (Hans Rudolph). Peak heart rate will be measured through a 12-lead electrocardiogram, and blood pressure and transcutaneous oxygen saturation will be measured after 5 minutes of rest in the sitting position, during the test, and shortly after termination. The Borg rating [50] of perceived exertion will be assessed at the end of the test. The VO_2_peak is defined as the highest oxygen consumption value sampled over a 30-second interval. The ventilatory threshold will be calculated using the ventilatory equivalent method, expressed as oxygen uptake (VO_2_). The minute ventilation (VE) and carbon dioxide responses during exercise will be used to calculate the VE/carbon dioxide uptake slope. The breathing reserve will be calculated using measured maximal voluntary ventilation (MVV) and the maximal VE using the following equation:

([MVV – VE]/MVV) × 100 **(1)**

The oxygen pulse will be calculated by dividing VO_2_peak (in mL) by the peak heart rate [51].

###### Isometric Strength

Isometric bench press and isometric leg extension will be performed using a custom-built strength ergometer (Gym 2000) with a strain gauge (US2A100 kg, Holtinger) and a custom-made amplifier. The ergometer is specially designed for testing children. In the bench press test, participants will be instructed to push an (upward) static bar with maximal effort for 5 seconds. The participants will lie on the ergometer with the bar height adjusted so that the elbow angle is 90° to get the upper arm in a horizontal position and the lower arm in a vertical position. The participants will be required to maintain the elbow angle throughout the duration of the trial. Each participant will perform at least three trials with 2 minutes of rest between each trial but will be allowed to continue with further trials as long as improvements are observed. The highest value of the trials will be used for later analysis. In the knee extension test, the participant will be seated at the short end of the ergometer bench with his or her back straight, arms down, and hands lightly gripping the seat. During contraction, both hip and knee joint angles will be flexed at 90° (180° will be fully extended), respectively. For the knee extension isometric test, the participants will also perform at least three trials with 2-minute rest between trials but will be allowed to continue with further trials as long as improvement is observed. The best result in each test will be used for later analysis. Hand-grip strength will be measured in the left and right hands (Baseline Life Hand Dynamometer) while the participant will be standing and with the arm extended and pointing down. Each hand will be measured 3 times, with alternating sides, starting with the right hand and approximately 30-second breaks between measurements. The best result for each hand will be used for later analysis.

###### Muscular Endurance

Muscular endurance will be tested using the 1-minute sit-to-stand (STS) test [52]. The participants will perform 1 test trial at least 20 minutes before the final test. The number of repetitions of standing up and sitting down from a chair within 1 minute in the final test will be recorded. In addition, the Borg rating [50] of perceived exertion will be assessed at the end of the test. The test will be performed on a height-adjustable chair to ensure a 90° knee angle. The 1-minute STS test showed high reliability and good criterion-related validity with other exercise capacity tests such as the 6-minute walk test or stair climbing [53-56].

###### Countermovement Jump

All jumps will be performed on a portable force plate (FP4; HUR-Laboratories Oy). Hands will be placed on the hips throughout the test, and the angular displacement of the knees will be standardized so that the participants are instructed to squat down until the knees are bent at approximately 90° and then immediately jump vertically as high as possible, landing back on the force plate on both feet at the same time. The jump height and power will be measured [57]. The best result of at least three attempts will be recorded; however, the participants will be allowed to continue to jump as long as improvements are made. There will be 1 minute of rest between the trials.

###### Blood Volume and Hemoglobin Mass

A known carbon monoxide (CO) dose of approximately 1.2 mL/kg body mass will be administered and rebreathed for 2 minutes. Capillary fingertip blood samples will be collected before the start of the test and 7 minutes after the administration of the CO dose. Both blood samples will be measured a minimum of 5 times to determine the percentage of carboxyhemoglobin using an OSM3 Hemoximeter (Radiometer). Hemoglobin mass will be calculated from the mean change in percentage of carboxyhemoglobin before and after rebreathing CO [58]. Blood volume, plasma volume, and red cell volume will be calculated from hemoglobin mass using venous hemoglobin and venous hematocrit according to Burge and Skinner [59] and Heinicke et al [60].

###### Lung Function

Lung function will be measured using the maximal expiratory flow volume loops. Forced expiratory volume in 1 second, forced vital capacity (FVC), maximal expiratory flow at 50% of FVC (MEF_50_), peak expiratory flow, and the ratio of forced expiratory volume in 1 second to FVC will be recorded. Lung volumes (total lung capacity and residual volume) and airway resistance will be measured using standardized whole-body plethysmography. Diffusion capacity will be measured using the single-breath method, and the result will be adjusted for serum hemoglobin levels. All measurements are according to the American Thoracic Society/European Respiratory Societies guidelines. The Global Lung Function Initiative reference equations will be used, and the outcomes will be expressed as absolute values, percent predicted, and *z* scores and the lower limit of normal.

###### Cardiac Examination

A full functional echocardiography protocol, including 2D, color, pulsed, continuous, and tissue Doppler registrations, will be performed using a GE Vivid E95 ultrasound machine (GE Healthcare) with 4- and 5 MHz 2D probes (4Vc-D or M5Sc). 3D volumes will be acquired as multi-beat fusions with breath hold. The frame rate for 2D images will be kept between 50 and 90 frames per second for 2D strain measurements. Automatic image optimization will be used as the default setting, supplied by manual optimization, as required. Parasternal, apical, selective right ventricular apical, and subcostal views will be applied. For clinical use, left ventricular ejection fraction, as calculated by the Simpson biplane method, fractional shortening by m-mode (MediMatic Compacs software), and peak systolic left ventricular global longitudinal strain (GE EchoPac software), will be reported in addition to any other positive findings of clinical significance. Additional research-based measurements will be performed as the mean value of 3 consecutive cardiac cycles. Left ventricular 4D volumes will be measured using a semiautomatic method (EchoPac Auto-LVQ).

###### Neurological Evaluation

Detailed patient history and clinical neurological examination will be performed. The symptoms and findings relevant for polyneuropathy and neuropathic pain will be scored according to the Toronto Neuropathy Symptom Score, Douleur Neuropathique 4, and Neupsig criteria for neuropathic pain [61-63]. Standard nerve conduction studies on three motor nerves (ulnar, peroneal, and tibial), four sensory nerves (ulnar, radial, superficial peroneal, and sural), and one mixed motor or sensory nerve (tibial plantar nerve) will be performed. Assessment of small fiber function will be conducted by quantitative thermal testing (cold perception threshold and warmth perception threshold) on the hands and feet. All measurements will be performed on the right extremities. The temperature will be maintained at >30 °C in feet and >32 °C in hand. Polyneuropathy will be diagnosed and classified according to the criteria suggested by Tesfaye et al [64] as possible, probable, or confirmed polyneuropathy (clinical grading) and according to the Tesfaye neurophysiological criteria (stadium 0, 1a, 1b, 2a, and 2b), which includes subclinical polyneuropathy.

###### Anthropometry and Maturity

Body weight will be measured using a digital scale (SECA 876 GmbH and Co), with participants wearing light clothing and no shoes. Height will be measured using a stadiometer (SECA 217 GmbH and Co). Body composition will be assessed by dual-energy x-ray absorptiometry (Lunar iDXA GE Healthcare using the enCORE software Version 14.10.022). Participants will be scanned from head to toe in a supine position, providing values for total lean tissue (kg), fat mass (kg), bone mineral content, and bone mineral density. In WP 2, the puberty stage of survivors of childhood cancer will be assessed clinically by a medical physician at the first visit in Norway. In addition, all participants (survivor of childhood cancer and controls) will self-assess their puberty stage using a modified Tanner method [65].

###### Muscle Thickness

Muscle thickness and pennation angle of the musculus vastus lateralis and thickness of the musculus biceps brachii will be measured by B-mode ultrasound using a linear-array transducer (50 mm, 5-12 MHz, HD11XE, Philips Ultrasound, revision 2.0.3). The sites for the measurements will be on one-third of the humerus length measured from the lateral epicondyle for the musculus biceps brachii and on 40% of the femur length measured from the lateral epicondyle for the musculus vastus lateralis.

###### PA Measures

Whenever the time between participation in WP 1 and recruitment in WP 2 exceeds 3 months, PA will be remeasured by accelerometers as described above (WP 1).

##### Data Management

Study data will be collected and managed using REDCap (Research Electronic Data Capture) electronic data capture tools hosted at the Norwegian School of Sport Sciences [66,67]. REDCap is a secure, web-based software platform designed to support data capture for research studies. Data from all sites will be entered by the study sites independently in REDCap.

##### Power Calculation and Statistical Analyses WP 2

Sample size calculations and power assessment were based on VO_2_peak. On the basis of results from international studies, we expect to find a difference of 10% between survivors of childhood cancer and controls [22]. To detect a difference of 10% in VO_2_peak between survivors and healthy controls, with a statistical strength of 80%, the study needs to include 23 survivors of childhood cancer. The same number of patients are needed to get the same statistical power for the strength tests assuming a difference of 10%. Owing to heterogeneity between diagnostic groups and the need for subgroup analysis (diagnosis, age, and gender), we plan to include 150 survivors as we expect the group size to be as small as 30 participants.

We will use descriptive statistics to present the current PF, physiological determinants of PF, and PA of survivors of childhood cancer compared with controls. We will use multilevel (mixed) linear models to compare PF and PA between survivors of childhood cancer and controls and include an interaction term (for instance, age group, gender, and diagnosis) to identify differences in physiological determinants between survivors of childhood cancer and controls. All analyses will be adjusted for the study site by including sites as random intercepts in the mixed models. Where available and depending on the research question, the fitness outcomes will be transformed into *z* scores according to the norm data. Depending on the outcome, we will perform univariable and multivariable mixed logistic, linear, or ordinal regression models to identify clinical and physiological factors associated with PF in survivors.

#### WP 3

The outcomes of WP 3 include facilitators of and barriers to PA. Norway (Oslo and Bergen) and Denmark (Copenhagen) will participate in WP 3.

##### Theoretical Framework and Procedures

The World Health Organization’s International Classification of Functioning, Disability, and Health for Children and Youth (ICF-CY) illustrates the interrelationships between body functions and structural impairments (eg, late effects), activity limitations, and restricted ability to participate within an individual’s context of environmental and personal factors to predict outcomes (eg, functioning and health) [68]. Therefore, the ICF-CY is a suitable biopsychosocial framework to systematically identify potential barriers to and facilitators of PA among young survivors of childhood cancer. Inspired by the ICF-CY model, we will explore facilitators of and barriers to PA through in-depth interviews with young survivors of childhood cancer and their parents. A semistructured interview guide will be developed based on current knowledge of barriers to and facilitators of PA and inspired by the ICF-CY framework (Multimedia Appendix 1) to be used at all 3 sites. The interviews will follow the guide and last approximately 15 to 60 minutes. All interviews will be audio recorded and transcribed ad verbatim.

##### Data Analysis WP 3

NVivo software will be used for data management and analysis [69]. Analysis will be conducted in parallel with data collection to ensure that any new issues of interest arising are explored in subsequent interviews. Interviews from survivors and parents will be analyzed separately. All data will be analyzed according to the principles of thematic analysis [70,71]. After familiarization with the initial interviews, at least two researchers will analyze the data line by line, creating codes reflecting the contents of each utterance to ensure objectivity [72,73]. Any disagreements regarding the codes will be discussed with the project group and settled. For analytical rigor, codes will be used and discussed by all members of the research team to develop a codebook that will be used in all interviews for all sites. We will follow the COREQ (Consolidated Criteria for Reporting Qualitative Studies) guidelines for conducting and reporting qualitative research [74].

#### WP 4

##### Overview

WP 4 aims to develop and feasibility test a sustainable and safe PA intervention for survivors of childhood cancer based on the results obtained in WPs 1 to 3. Norway, Finland, and Germany will participate in WP 4. The study will comprise two study visits (baseline and after 6 months), including self-reported questionnaires, interviews, measurement of PA, and PF tests (Table 1; Table S1 in Multimedia Appendix 1). In between, the survivors will participate in a personalized PA intervention, followed by central and local coaches.

In specific, we aim to do the following:

1. Describe the adherence to and acceptability, satisfaction, and safety of the personalized PA intervention
2. Test the involvement of local structures (parents, school, teachers, peers, community and school nurses, and sports clubs) to improve the PA of survivors
3. Assess the effect of the intervention on PA, attitude toward PA, QoL, fatigue, and PF
4. Explore the experiences of survivors of childhood cancer and their parents of participating in the PA intervention
5. Explore the central coaches’ experiences providing the intervention and collaborating with local structures

##### Intervention

The exercise intervention will last 6 months and starts with a personal motivational interview (MI) session at the baseline study visit. For each survivor, a central study coach is appointed who performs the MI and, together with the participant, defines a personal target of duration and intensity of PAs per week. The MI technique is a communication method that involves enhancing a patient’s internal motivation to change [75]. During this session, local structures (local coaches) will be identified that can help and support the survivor to reach their PA goals and maintain a more active lifestyle after the intervention. On the basis of this assessment, individualized PAs are defined and implemented into a survivor’s daily life. Each survivor will get a written personalized plan at the end of the session and a Polar watch (Polar Global) to track their behavior and provide immediate feedback on their achievements.

The central study coach will follow up with the survivor and local coaches with a phone call based on a predefined schedule of decreasing frequency over time (Table S1 in Multimedia Appendix 1). During the follow-up phone calls, the coach uses MI-inspired techniques and a standardized guideline to discuss adherence, motivation, progress, and possible problems. The training program can be adapted, and solutions for solving problems will be searched. The central coach will also follow the PA behavior in real time based on the Polar watch and set up an additional follow-up contact if the survivor struggles to reach his or her activity goals. The central coaches will be trained in the use of MI techniques to be used in the initial and follow-up conversations with survivors.

##### Assessments

Table S1 in Multimedia Appendix 1 gives an overview of all assessments in WP 4.

##### Feasibility Parameters

###### Recruitment

The number of contacted and recruited survivors with reasons for nonrecruitment will be recorded by the study nurses at each site.

###### PA Parameters

PA will be measured by an accelerometer as described for WP 1 at baseline and after 6 months. In addition, the PA will be tracked continuously with the Polar watch (Polar Global). At each follow-up discussion with the central coaches, the survivors will be asked about their participation in the agreed PA program and, if applicable, reasons for nonparticipation.

###### Compliance (Adherence and Retention)

Compliance with the PA intervention will be assessed based on the PA measurements described above. In addition, we will record the number of contacts of each survivor with the central and local coaches and the number of missing information at each study time point. Dropouts and reasons for dropout will be assessed by the study nurses.

###### Safety

Safety and adverse events will be assessed by the central coaches during follow-up calls.

###### Subjective Rating of the Intervention and Its Motivational Features

At the end of the intervention, the survivors will give their subjective rating of satisfaction with participation in and intended sustainability of the PA intervention. The questions will be developed specifically for this study.

###### Attitude Toward PA (Barriers, Perceived Physical Competence, Perceived Enjoyment, and Motivation)

Attitudes toward PA will be assessed at baseline and at 2 and 6 months. The same questions as in WP 1 will be used to assess *barriers to PA*. *Perceived physical competence* (3 items) and *perceived enjoyment* (5 items) will be measured by items from the *Children’s Attraction to PA* scale [76-78]. *Motivation toward PA* will be measured using the Behavioral Regulation in Exercise Questionnaire 2 [79].

###### QoL and Fatigue

At baseline and 6 months, survivors will fill in the same questionnaires as in WP 1 to assess QoL and fatigue (only self-reported version and no parent module).

###### PF Assessment

PF will be assessed during the study visits at baseline and after 6 months. All sites will perform a CPET and 1-minute STS test according to the same protocol as described for WP 2. In addition, Oslo and Bergen will perform the same isometric strength tests (knee extension and chest press) as in WP 2. Turku will perform the same knee extension test as WP2 but on its own device as well as a dynamic upper extremity lifting test (standing with 5 kg dumbbells for women and 10 kg for men; number of repetitions are reported). Essen will perform the same isometric knee extension and chest press as in WP2 but on their own devices.

##### Qualitative Interviews With Survivors, Parents, and Central Coaches

Feasibility, acceptability, sustainability, and satisfaction with the PA intervention will also be assessed by qualitative in-depth interviews in a subsample of survivors and their parents from the Oslo site at the 6-month visit (8-15 survivors and their parents until information saturation is reached). Furthermore, the central coaches will participate in a focus group interview to explore their views on feasibility, acceptability, and satisfaction. The interview guides are provided in Multimedia Appendix 2.

##### Data Management

All self-reported questionnaires will be answered electronically either on the study tablet PCs during the study visits (baseline and 6 months) or from home via a personal link (2 months). The questionnaires will be linked to the TSD secure data server at the University of Oslo, where the data will be stored. Device-measured data (Actigraph and Polar watch [Polar Global]) will be downloaded and added to the folder on the TSD. Information assessed by the study nurses, central coaches, and assessors of the physical testing will be collected in Microsoft Excel files and stored on TSD. All information will then be merged into a master data file using the statistical software STATA (version 17 or newer; StataCorp LLC).

##### Data Analyses WP 4

###### Quantitative Data

As this is a feasibility study, we will mainly use descriptive statistics to present the study results. We will use numbers and proportions to describe categorical variables and means, SDs (or median and IQR if the data are skewed), and ranges to describe continuous variables. *P* values will be calculated based on appropriate univariable and multivariable models depending on the outcome and adjusted for the study site by introducing a random intercept (mixed model). *P* values are interpreted as flagging trends and not as hypothesis tests.

###### Qualitative Data

Qualitative content analyses of interview transcripts will be performed [70].

### Ethics Approval

The PACCS study has been approved by the Norwegian Regional Committee for Medical Research Ethics (WP 1,3,4: 2016/953 and WP 2:2018/739), the Data Protection Officer at Oslo University Hospital, and by the equivalent institutions in Finland, Denmark (file. H-19032270), Germany, and Switzerland (Ethics Committee of Northwestern and Central Switzerland; project ID: 2019-00410). Approvals from the regional ethical committees for medical and health research at all sites and the project owner institution are the assumptions for the implementation of the project. As most of the participants will be too young to provide valid informed consent, they will receive written and verbal information about the studies adapted to their developmental stage. Parents or legal guardians will provide written consent on their behalf. School nurses, physical education teachers, and other relevant parties participating in WP 3 will provide informed consent in writing. This protocol is written according to the SPIRIT (Standard Protocol Items: Recommendations for Interventional Trials) guidelines [80].

## Results

Research activity for this study commenced in November 2016 with the piloting of questionnaires and recruitment to WP 1 at the Oslo, Norway site. The project received funding from the Research Council of Norway for the period from June 2018 to June 2022. International collaboration was established after June 2018. Data collection at all sites was initiated by October 2020 for WP 1 and concluded on January 2021, with 517 survivors of childhood cancer included. Data cleaning and analysis are currently underway. A total of articles are under preparation for publication. Data collection for WP 2 was initiated in January 2019 and concluded in December 2020, with 157 survivors of childhood cancer and 113 controls included. Data cleaning and analysis are currently being conducted. A total of 6 articles are planned based on the WP 2 data set. Data collection for WP 3 was initiated in January 2018. Patient and parent interviews were concluded in July 2020, with 63 survivors of childhood cancer and 68 parents. Interviews with local stakeholders were initiated in January 2020 and concluded in October 2020, with a total of 18 participants interviewed (teachers, special needs teachers, and school nurses). Data from the patient and parent interviews have been analyzed and are currently being written for publication. A total of 2 articles have been published as of January 2022 [81,82], and 2 more are under preparation for publication. Data from interviews with local stakeholders are currently being analyzed, and 1 article is planned. WP 4 recruitment was initiated in May 2021 and is still ongoing as of January 2022. The study is planned to be completed by July 2022. As of January 2022, 23 survivors of childhood cancer have been included, of which 11 have completed the intervention. Detailed plans for data analyses for WPs 1 to 4 are described in the "Assessments, Power Calculations, and Statistical Analyses" section.

## Discussion

### Principal Findings

Survivors of childhood cancer represent a constantly growing population in society with high morbidity [4], premature mortality [83], and increased uptake of social benefits [84]. Owing to their disease and treatment exposure, they are at risk of chronic medical conditions such as cardiovascular and pulmonary diseases, secondary cancers, metabolic syndrome and obesity, osteoporosis, fatigue, neurocognitive complaints, and psychological distress, all of which can negatively affect PF and function and, thereby, the survivors’ ability to participate in everyday and social activities [3,4,8]. Options for preventing late effects are currently limited; however, a few small-scale intervention studies indicate promising effects of PA on promoting physical functioning, health, and QoL [10-13]. However, important knowledge gaps and methodological shortcomings remain, including heterogeneity of the samples, lack of controls, quality of assessment methods, and outcome measures used, limiting the generalizability of the results [17]. This applies to survivors of childhood cancer in general, especially for young survivors of childhood cancer aged <18 years.

The PACCS study will generate high-quality, generalizable, objective, and robust data on PA and PF in young survivors of childhood cancer compared with controls, as well as on physiological, psychological, and social factors associated with PA and PF in young survivors of childhood cancer. These data are crucial to establish the extent of the need for PA interventions for young survivors of childhood cancer and for the development and testing of evidence-based, safe, and efficacious PA interventions. Moreover, the data generating WPs and the intervention development will follow a behavioral science approach, supported by the theoretical framework of the ICF-CY, to strengthen their methodological rigor [85].

The resulting intervention will make use of and test behavioral change strategies (MI) for feasibility as a means of tailoring PAs and goals to the individual survivors, with adherence monitored using activity-tracking watches. This will contribute to overcoming barriers to PA and low adherence rates, thereby potentially increasing the sustainability of such interventions. Crucially, sustainability of the intervention is boosted by anchoring it locally in the survivor’s home environment, in collaboration with key school, health, and sports activity personnel to maintain focus on PA beyond the intervention period.

PA is currently not a part of routine follow-up care in many countries, and non–evidence-based PA programs are only sporadically offered to survivors of childhood cancer. Thus, the international, multidisciplinary nature of the PACCS consortium, representing 6 European pediatric oncology institutions, will aid the harmonization of PA programs and counseling. Therefore, the project results may improve the current clinical practice.

Promoting regular moderate to vigorous levels of PA in survivors of childhood cancer is likely to reduce cardiovascular morbidity [86] and mortality [14,87-89], osteoporosis, and fatigue and increase psychological well-being. Moreover, as PA is an important determinant of PF, both PA and PF are essential for gaining developmental skills in children and adolescents. Ensuring sufficient levels of PA in young survivors of childhood cancer is likely to have long-reaching benefits for their functioning and, therefore, the opportunity to be active and participating members of society, including maintaining friendships and participating in sports and recreational activities. As such, systematic PA rehabilitation programs have the potential to reduce the burden of survival on individual survivors, as well as reduce health care and social costs for the survivors of childhood cancer.

### Conclusions

Adolescence provides a *golden period* of life for the establishment of healthy behaviors and is, therefore, the prime target for intervention [27,42,90]. Our intention is to design a rehabilitation program that uses already existing local expertise and infrastructure. Moreover, if successful, the results of the observational study and feasibility intervention may be extrapolated to other groups of adolescents with chronic health problems; for example, adolescents with congenital heart defects or chronic pulmonary diseases. Thus, the project may benefit larger patient groups.

## Appendix

Multimedia Appendix 1 Schedule of enrollment, interventions, and assessments within Physical Activity and Fitness in Childhood Cancer Survivors work package 4.

Multimedia Appendix 2 Interview guides work packages 3 and 4.

Multimedia Appendix 3 Peer review report from project application process by the Research Council Norway.

## Abbreviations

CO: carbon monoxide
COREQ: Consolidated Criteria for Reporting Qualitative Studies
CPET: cardiopulmonary exercise test
cpm: counts per minute
FVC: forced vital capacity
ICF-CY: International Classification of Functioning, Disability, and Health for Children and Youth
MI: motivational interview
MVV: maximal voluntary ventilation
PA: physical activity
PACCS: Physical Activity and Fitness in Childhood Cancer Survivors
PedsQL: Pediatric Quality of Life Inventory
PF: physical fitness
QoL: quality of life
REDCap: Research Electronic Data Capture
REPAC: Relevance of Physical Activity Contexts in the Everyday Life of Adolescents
SDQ: Strength and Difficulties Questionnaire
SPIRIT: Standard Protocol Items: Recommendations for Interventional Trials
STS: sit-to-stand
VE: minute ventilation
VO_2_: oxygen uptake
VO_2_peak: peak oxygen uptake
WP: work package
TSD: Services for Sensitive Data
