# Supplementary material for: The Physical Activity and Fitness in Childhood Cancer Survivors (PACCS) Study: Protocol for an International Mixed Methods Study
Source: JMIR Res Protoc. 2022 Mar 8;11(3):e35838. doi: 10.2196/35838 (PMC8941432; doi:10.2196/35838)
Supplement: Multimedia Appendix 1 [file resprot_v11i3e35838_app1.docx]

|  | | **STUDY PERIOD** | | | | | | | | | | | | |
| --- | --- | --- | --- | --- | --- | --- | --- | --- | --- | --- | --- | --- | --- | --- |
|  | | Enrolment/ baseline | Intervention | | | | | | | | | | | Close-out |
| **TIMEPOINT** | |  | 2 w | 4 w | 6 w | 8 w | 10 w | 12 w | 14 w | 16 w | 18 w | 20 w | 22 w | 6 mt |
| **ENROLMENT:** | |  |  | | | | | | | | | | |  |
|  | Eligibility screening | x |  |  |  |  |  |  |  |  |  |  |  |  |
|  | Contact and information | x |  |  |  |  |  |  |  |  |  |  |  |  |
|  | Informed consent | x |  |  |  |  |  |  |  |  |  |  |  |  |
| **INTERVENTION:** | |  |  | | | | | | | | | | |  |
|  | Physical activity motivational interview and counselling | x |  |  |  |  |  |  |  |  |  |  |  |  |
|  | Physical activity & exercise intervention |  |  |  |  |  |  |  |  |  |  |  |  |  |
|  | Coaching contacts from central coach^a^ |  | x | x |  | x |  | x |  |  | x |  |  | x |
|  | Support from local structures^c^ |  |  |  |  |  |  |  |  |  |  |  |  |  |
|  | Motivational feedback from Polar watch |  |  |  |  |  |  |  |  |  |  |  |  |  |
| **ASSESSMENTS:** | |  |  | | | | | | | | | | |  |
| **Physical activity** | |  |  |  |  |  |  |  |  |  |  |  |  |  |
|  | Continuous real-time tracking of PA with Polar watch |  |  |  |  |  |  |  |  |  |  |  |  |  |
|  | Accelerometery (1 weak wear time) |  |  |  |  |  |  |  |  |  |  |  |  |  |
| **Feasibility parameters** | |  |  | | | | | | | | | | |  |
|  | Compliance (based on PA tracking by polar and central coach follow-ups^b^) |  | x | x |  | x |  | x |  |  | x |  |  | x |
|  | Involvement of local supporters (central coach follow-ups^b^) |  | x | x |  | x |  | x |  |  | x |  |  | x |
|  | Safety: fatigue and pain VAS (central coach follow-ups^b^ and self-reported questionnaires) | x | x | x |  | x |  | x |  |  | x |  |  | x |
|  | Safety: assessment of adverse events (central coach follow-ups^b^) |  | x | x |  | x |  | x |  |  | x |  |  | x |
|  | Perceived enjoyment while doing physical activities (questionnaire) | x |  |  |  | x |  |  |  |  |  |  |  | x |
|  | Subjective rating of the motivational features (questionnaire) |  |  |  |  |  |  |  |  |  |  |  |  | x |
|  | Subjective rating of satisfaction, participation and sustainability (questionnaire) |  |  |  |  |  |  |  |  |  |  |  |  | x |
|  | Qualitative interviews to discuss all of the above mentioned feasibility outcomes |  |  |  |  |  |  |  |  |  |  |  |  | x^d^ |
| **Attitude towards physical activity** | |  |  | | | | | | | | | | |  |
|  | Barriers towards PA, motivation towards PA, perceived competence towards PA (all self-reported questionnaires) | x |  |  |  | x |  |  |  |  |  |  |  | x |
|  | Qualitative interview (to discuss all of the above outcomes) |  |  |  |  |  |  |  |  |  |  |  |  | x^d^ |
| **Physical fitness** | |  |  | | | | | | | | | | |  |
|  | Maximal performance test on treadmill | x |  |  |  |  |  |  |  |  |  |  |  | x |
|  | Strength test (isometric bench press and leg extension) | x |  |  |  |  |  |  |  |  |  |  |  | x |
|  | 1-min-sit-to-stand-test | x |  |  |  |  |  |  |  |  |  |  |  | x |
| **Quality of life and fatigue** | |  |  | | | | | | | | | | |  |
|  | Quality of life questionnaire | x |  |  |  |  |  |  |  |  |  |  |  | x |
|  | Fatigue questionnaire | x |  |  |  |  |  |  |  |  |  |  |  | x |
| **Personal information** | |  |  | | | | | | | | | | |  |
|  | Socio-demographic characteristics | x^e^ |  |  |  |  |  |  |  |  |  |  |  |  |
|  | Cancer-related information | x^e^ |  |  |  |  |  |  |  |  |  |  |  |  |

^a^ The central coach will add additional contacts with the survivor if needed depending on the PA behaviour observed in the real-time tracking with the Polar watch.

^b^ The central coaches ask standardises questions during the follow-ups based on a form developed for the study. The answers to the questions are noted on the form and stored for analysis at the end of the study.

^c^ The interval and intensity of the support from the local structures will be individualised based on the survivor’s preferences and availability of potential local coaches. Potential local coaches include: teachers, community nurse, school nurse, peer/buddy, trainer of a local sports club.

^d^ The qualitative interview will be done in a heterogenic subsample of survivors and parents in Oslo only (N=8-15 survivors and parents until information saturation is reached). The interview will be performed in first the survivor only and then the survivor and parent together and last 25-45 minutes. A focus group interview will be performed with all the central coaches of the study site Oslo.

^e^ The information will be available from the general PACCS database.

**Reference list**

1. Lien N, Bjelland M, Bergh IH, Grydeland M, Anderssen SA, Ommundsen Y, et al. Design of a 20-month comprehensive, multicomponent school-based randomised trial to promote healthy weight development among 11-13 year olds: The HEalth In Adolescents study. Scandinavian Journal of Public Health. 2010;38(5_suppl):38-51.

2. Dalene KE, Anderssen SA, Andersen LB, Steene-Johannessen J, Ekelund U, Hansen BH, et al. Cross-sectional and prospective associations between physical activity, body mass index and waist circumference in children and adolescents. Obes Sci Pract. 2017;3(3):249-57.

3. Erdvik IB, Haugen T, Ivarsson A, Säfvenbom R. The Temporal Relations of Adolescents’ Basic Need Satisfaction in Physical Education and Global Self-Worth. Journal of Sport and Exercise Psychology. 2020;42(6):480-9.

4. Samdal O, Mathisen FKS, Torsheim T, Diseth ÅR, Fismen A-S, Larsen TMB, et al. Helse og trivsel blant barn og unge. Resultater fra den landsrepresentative spørreundersøkelsen «Helsevaner blant skoleelever. En WHO-undersøkelse i flere land». 2016.

5. Helsedirektoratet. Ungkost 2000 - Landsomfattende kostholdsundersøkelse (nettdokument). . <https://www.helsedirektoratet.no/rapporter/ungkost-2000-landsomfattende-kostholdsunderokelse>: Helsedirektoratet; 2002.

6. Varni JW, Seid M, Rode CA. The PedsQL™: Measurement Model for the Pediatric Quality of Life Inventory. Medical Care. 1999;37(2):126-39.

7. Varni JW, Burwinkle TM, Katz ER, Meeske K, Dickinson P. The PedsQL™ in pediatric cancer. Cancer. 2002;94(7):2090-106.

8. Goodman R. The Strengths and Difficulties Questionnaire: A Research Note. Journal of Child Psychology and Psychiatry. 1997;38(5):581-6.
