# Supplementary material for: The Physical Activity and Fitness in Childhood Cancer Survivors (PACCS) Study: Protocol for an International Mixed Methods Study
Source: JMIR Res Protoc. 2022 Mar 8;11(3):e35838. doi: 10.2196/35838 (PMC8941432; doi:10.2196/35838)
Supplement: Multimedia Appendix 2 [file resprot_v11i3e35838_app2.docx]

**Multimedia Appendix 2 Interview guides WP 3 and 4**

**Interview guide, PACCS WP 3**

**Survivors**

Opening Questions:

What can you tell me about yourself? Promts:

- How old are you, what grade/class are you in, what do you like to do in your spare time?

- What do you remember about the time when you were sick?

- Do you remember how your physical form /fitness were then?

We are interested in knowing how it is for you to exercise, or to be physically active after completed cancer treatment.

1. What does the week look like- what are you doing?

• Is it difficult to exercise / be active now? (Tired / not so strong anymore / hard to hang out with your friends?)

• Are there enough leisure activities/ options where you live?

• What do you do with your friends?

2. What kind of exercise do you like?

• Why do you like it?

• How do you get to your activities/exercise? (Must anyone have to remind you to go?)

• What makes you perform physical activity /exercise /workout when you do not want to?

• How do you experience Physical Education at school?

3. Is there something that makes it difficult for you to exercise? If yes: What are you doing then?

• Are you as physical active as you want to be?

• Are you afraid to push yourself too much / too hard?

4. Have you talked to someone about these topics, or found the solution out all by yourself?

• Do your friends know that you have had cancer?

• Do you know someone with the same experiences as yourself? (peers with cancer)

5. What kind of leisure activities do your friends / siblings / parents participate in?

6. Can you explain why it is important to participate in physical activity?

7. If you had not had cancer, do you think that anything had been different for you?

8. Is there anything the hospital / doctors / nurses / physiotherapists / school / school nurse did or could have done to help you to be physically active?

9. Do you have any advice for other children/teens for what to do when they are finished with treatment to be physically active?

10. Is there anything else you want to say something about

Thank you for taking part in the interview

**Interview guide - Parents - PACCS – WP 3**

Opening Questions:

What can you tell me about your family?

Can you tell me about the time after your son/daughters cancer?

1. What activities is your son/daughter attending now?

2. How did you / the child decide on these activities?

3. How is the physical education at school?

4. Who makes sure that the child shows up for physical activities / leisure activities?

5. Are you ever worried that your child is pushing himself/herself too hard?

6. Do you notice any difference in the child in relation to peers in physical activity?

7. Are you talking about this in your family?

8. Does your child know that you are worried?

9. Do you think that your son/daughter is concerned about this?

10. Is there something that makes it difficult for your child to exercise?

11. Is it easy to motivate the child to exercise?

12. Are you interested in physical activity in your family?

13. What do you think could have been done to make it easier for him /her to be active / exercise after completed treatment?

14. What advice do you have for other parents regarding how to motivate for physical activity after the child has completed treatment?

15. Is there anything else you would like to say something about?

Thank you for participating in the interview!

**Supplemental Appendix 2: interview guides, WP 4**

Adolescents:

WP 4 Interview guide - after the intervention.

Introduction:

Welcome and thank you for taking the time to participate in this interview. We will ask you some questions about the activity study that you have participated in these 6 months and we would like to hear your experiences as a participant. We would like you to tell as much and as freely as possible along the way. I will ask some follow-up questions or ask you to elaborate. We will use an audio recorder to record the conversation so that we remember everything later. The interview will last about 1 hour. You can withdraw from the interview at any time and then have the right to have all data and information collected about you deleted.

"Now I would like your honest feedback on this “Activity study” or intervention as we also call it"

1. Why did you want to join when you were asked 6 months ago? (Follow-up: how physically active were you then? what did you like to do when being physical activity, why did you want to participate (motivation), expectations for participation?).

2. When it comes to your participation in the study, we are curious about what you liked and did not like about participating? Tell me a little about it. (follow-up: What do you think about the type of activities you did, What was fun and what was not so fun? The frequency of the assessments, the help you received from local coach / local staff / friends / family? What do you think about the length of the study, and the different parts of the intervention (eg testing, interviews, questionnaires, duration)?

3. When you chose to join this study, was there anything special you wanted to achieve? (follow-up: Were there things you thought participating in this study would help you achieve? If so, please explain. How were / were your expectations not met?)

4. Was anything worrying about participating? (For example, pushing yourself too hard, thinking that it would be difficult or too time consuming / that it would make you even more tired etc?)

5. What type of training did you do during the project? - can you tell us a bit about your training program? (follow-up: what has happened to your physical form along the way? What goals have you had, how has it been to follow through with the program? – can you tell about the training plan ?, Polar clock? Any positive changes in your own fitness?, mastery belief, importance of training ?, what has been important in relation to motivation to be physically active ?, what has worked well?)

6. What helped you to be motivated to train throughout the intervention? (follow-up: POLAR watch, central / local coach, your parents, your friends, others? Goals / personal goals and inner motivation?)

7. If you think about the time before you participated in this study and how you felt then, and how it is now, have you noticed any changes in your life? (follow-up: fatigue, sleep, pain, increased function and ability to participate in social or other activities, school, etc.)

8. What was it like talking to the coach (use name) from the sports college? (follow-up: What did you talk about? How do you think it was to talk to the coach the first time? How did you experience the telephone conversations? What do you think the coach's role has been? What do you think about how the conversations were set up? Suggestions for improvement / change if later studies?)

9. How did you experience your local coach? (who was it? how was it organized, what worked well, what about in the future? suggestions for improvements?)

10. What was it like to include a friend to train with? / What significance do your friends have in relation to your activity and training? (follow-up: What do you think about further training and activity after the study? (Motivation, friends ?, what about the local support staff ?, what follow-up do you need after the intervention?)

11. Did your expectations change about what participating in the study could mean to you along the way? (follow-up: Do you want to participate in a study like this again? Why or why not? Would you recommend this program to a friend? Why or why not?)

12. What advice would you give to other young people who have completed treatment in relation to activity?

13. If the project is to continue - what do you think should be done differently, do you have any advice for us? / What can we do better next time?

12. Finally, what plans do you have for being active now that you have completed your study participation? (motivation, joy of training, friends, what about the local support staff ?, what follow-up do you need further?)

**WP 4 Interview guide focus group coaches - after the intervention.**

**Introduction:**

Such a discussion that you will now take part in is called a focus group interview. The purpose of such interviews is to shed light on a topic by seeking advice from people who have a lot of experience on the subject. Therefore, you will discuss and reflect together, and share your thoughts and experiences in relation to the training intervention and your role in WP 4. I will ask some questions along the way, but here there are no right or wrong answers. What we want are your thoughts and experiences in relation to the topic and that you discuss and reflect together.

Brief information about how we will proceed:

- It is desirable that everyone has the floor.
- The meeting will be recorded on audio recorders and the conversation will be printed in full text later. If the names of some people are mentioned during the conversation, these names will be removed when the audio recording becomes text.
- Since the conversation is recorded and will be printed in text afterwards, it is good if you try to speak one at a time.
- Everyone has been given a pen and paper so that you can write down key words if there is something you want to write down to remember later.
- What is said will be treated confidentially and we hope that you agree on a mutual duty of confidentiality within the group so that no one refers to things from our conversation so that others can be recognized.
- The meeting will last for about 1.5 hours.

Questions:

1. What do you think about being a coach in this intervention?

2. What do you think has been most positive about being involved (in relation to the young people, yourself)?

3. What do you think motivated young people along the way?

4. What has been challenging?

5. What do you think about the testing at the Norwegian School of Sport Sciences (NIH) before the intervention and the first meeting with the youth

6. What do you think about using motivational interviewing techniques - how did it go? - what could have been done differently in teaching/ using the MI method?

7. What was it like to keep in touch with the youth during the intervention?

8. How did you contact the local coach during the intervention - collaboration and roles?

9. How has the Polar clock been useful to you as a coach - proposed changes?

10. What type of follow-up did you receive from each other (coaches) during the period, how should this be done if the project is to continue

11. What qualities should one have as a coach? Who are relevant coaches?

12. What about time use?

13. What changes have you noticed in the young people from before to after the intervention

- what type of youth is this intervention suitable for?

14. What might be the goal of a new study?

15. What do you think about the results so far?

16. Some young people said they wanted more visits to the NIH - what do you think about that?

17. What do you think about the length of the intervention? What about the summer holiday in the middle of the intervention?

18. Some young people wanted closer follow-up when their motivation was low or they were not able to train - what do you think?

19. How did the collaboration with the parents go - what was their role in relation to you?

20. If the project is to continue - what do you think should be done differently?

21. What are the important factors for such an intervention to be successful in a large RCT study?
