# Supplementary material for: The Physical Activity and Fitness in Childhood Cancer Survivors (PACCS) Study: Protocol for an International Mixed Methods Study
Source: JMIR Res Protoc. 2022 Mar 8;11(3):e35838. doi: 10.2196/35838 (PMC8941432; doi:10.2196/35838)
Supplement: Multimedia Appendix 3 [file resprot_v11i3e35838_app3.pdf]

Prosjektvurdering - Karaktersammendrag

Prosjektnummer: 273500
Prosjekttittel: Physical Activity and Fitness among Childhood Cancer Survivors

	Vurderingskriterium	Karakter	Gjennomsnitt fra eksperter / panel
1	Vitenskapelig kvalitet	6	6.0
2	Prosjektleder og prosjektgruppen	6	6.0
3	Brukermedvirkning	A	7.0
4	Forventet nytteverdi	A	7.0
5	Gjennomføringsplan og ressursbehov	A	7.0
6	Nasjonalt samarbeid	A	7.0
7	Internasjonalt samarbeid	A	7.0
8	Formidling og kommunikasjon	A	7.0
9	Samlet vurdering fra fageksperten/panelet	6	6.0
10			
11			
12			
13			
14			
15			
	Hovedkarakter*	6	

Karakterskala: 7, 6, 5, 4, 3, 2, 1 (7 er best)
A, B, C (A er best)

*) Hovedkarakter er et uttrykk for hvor godt prosjektet oppfyller intensjoner og formål for søknadstypen.

Karakterene er en del av underlaget ved det besluttende organs behandling av søknaden.

Assessment of grant application submitted to the Research Council of Norway

Grant application

Project number	273500
Project title	Physical Activity and Fitness among Childhood Cancer Survivors
Project manager	Utne, Peder Heyerdahl
Project Owner	OSLO UNIVERSITETSSYKEHUS HF
Application Type	Researcher Project
Programme/Activity	God og treffsikker diagnostikk
Case officer	Karianne Solaas

Confirmation

By completing and submitting this form, I / we confirm the following (applies for the individual referee or the referee panel):

- I am /We are qualified to assess this application. See Regulations on Impartiality and Confidence in the Research Council of Norway.	Yes
- I/We have read and understood both the criteria I/we have been asked to use for assessing the application and the description of the scale of marks. The scale of marks is to be applied as an absolute scale, i.e. marks are to be determined for each grant application independently and not relative to other applications that the panel/referee is assessing.	Yes
- I/We understand and accept the guidelines for assessing applications for the Research Council of Norway. See Guidelines for referees/panels who assess applications for the Research Council of Norway.	Yes
- I am/We are qualified to conduct this assessment.	Yes

Summary of marks

Criterion	Mark
Scientific merit	6
The project manager and project group	6
User involvement	A
Anticipated benefits	A
Implementation plan and resource parameters	A
National cooperation	A
International cooperation	A
Dissemination and communication of results	A
Overall assessment of the referee/panel	6

Criteria

Scientific merit

How would you rank the project’s scientific merit?

This criterion gives an indication of the essential, fundamental aspects of the research project. The scientific merit of a project will be assessed in relation to the following points:
* Originality in the form of scientific innovation and/or the development of new knowledge.
* Whether the research questions, hypotheses and objectives have been clearly and adequately specified.
* The strength of the theoretical approach, operationalisation and use of scientific methods.
* Documented knowledge about the research front.
* The degree to which the scientific basis of the project is realistic.
* The scientific scope in terms of a multi- and interdisciplinary approach, when relevant.

This is a comprehensive and ambitious project that aims to characterize physical activity in childhood cancer survivors and understand whether this presents an opportunity to reduce longer term risks in this population. The work is very clearly described and the various work packages are logical and well justified. The investigators provide a clear rationale for the proposed work and experimental design and analysis plan(s) are carefully explained.

There is a clear need for more research in this area as highlighted in both the main research proposal document and in the anticipated benefit document. Past research in this area has been of a low quality and/or using small sample sizes. The commitment to undertake research on a much larger scale in multiple countries - and to undertake development work rather than prematurely jump straight to an intervention is another key strength.

Selected mark : 6 - Excellent

The project’s objectives, research questions and hypotheses are very clearly presented and are based on an excellently formulated and highly original project concept. The project is in the forefront of its field and will contribute to scientific innovation as well as generate important new knowledge. The project is of excellent quality, with no significant weak points. Publications in leading scientific journals in the field are highly likely.

The project manager and project group

How would you rank the qualifications of the project manager and project group?

This criterion gives an indication of the qualifications of the project manager and project group. The project manager and project group will be assessed in relation to the following points:
* Project management
* Expertise and experience within the field of research
* Publication record
* Experience with national and international collaboration on projects
* Experience with supervision of students and younger researchers
* The degree to which the project manager and project group are part of a research environment that has the competence and resources needed to ensure the success of the project

The project team that has been assembled is excellent and extremely well placed to conduct the proposed research. The international partners and collaborators are entirely appropriate. There is some mention of how the work will be managed in different countries, although a little more detail here would have been helpful.

Selected mark : 6 - Excellent
The project manager and/or research/project group is/are qualified at a high international level, has/have contacts within the foremost national and international research environments and will be able to play an important role in ensuring the success of the project.

User involvement

How would you rank the user involvement in the project?

This criterion is used to assess how well user perspectives and user involvement are incorporated into the project, or how well the lack of user involvement is explained. User involvement will be assessed based on the description of the following:
• Relevant users for the project;
• User involvement in the project planning;
• User involvement project implementation;
• User involvement in utilisation of project results.

The user involvement is extremely good.

Selected mark : A - Very good
The user perspective and user involvement are very well incorporated in all phases of the project.

Anticipated benefits

How would you rank the benefits of the project?

This criterion is used to assess how great of an impact and benefit the project results will have for the users, the research field and society at large. The benefits of the project will be assessed in relation to the following points:
- Benefit for the users and the research field;
- Benefit for society at large;
- Framework for the realisation of the benefits.

The long term anticipated benefits from this research are very strong based on the assumption that it will be possible to develop an intervention that will be scalable and effective.

Selected mark : A - Significant
The project has significant benefits.

Implementation plan and resource parameters

How well-suited are the implementation plan and resource parameters in relation to the project?

This criterion gives an indication of whether the plan for project implementation is satisfactory, and whether the planned use of resources in the project is well-suited for the tasks in the project, based on assessment of the following elements:
* Plans for project implementation, including breakdown into work packages/sub-projects, milestones and deliverables.
* Need for personnel resources, as listed in terms of work time distributed by work packages, sub-projects or milestones.
* Need for other resources (such as equipment, data collection, field work), distributed by work packages/sub-projects or milestones.

The assessment is not to be linked to any scientific risk.

The implementation plan appears logical and, with the various collaborators, the project appears realistic within the stated time frame.

Selected mark : A - Very good
The project plan and planned use of resources are very clearly described and are well-suited to the tasks in the project.

National cooperation

To what degree will the project promote national cooperation?

This criterion gives an indication of the extent to which the project will make use of national research expertise and help to promote national network-building.

The project involves various partners drawn from across Norway and each partner has a clearly defined role or contribution.

Selected mark : A - Very good
The project will make comprehensive use of national research expertise and will contribute greatly to promoting national network-building.

International cooperation

How would you rank the international cooperation set out for the project?

This criterion gives an indication of the extent and quality of the international cooperation activities set out for the project.

There is a strong international element to this project and the various international partners are integral to the success of the project. They are also the right partners for this project.

Selected mark : A - Very good
The international cooperation activities set out for the project have a wide scope and are of high quality.

Dissemination and communication of results

How would you rank the quality of the dissemination and communication plans?

This criterion gives an indication of the quality of the dissemination and communication plans for the project. Dissemination and communication of results will be assessed in relation to the following points:
* Plans for scholarly publication, dissemination and other communication activities.
* Plans for popular science dissemination and communication activities vis-à-vis the general public as well as users of the project results, including planned use of channels and measures.
* Plans for ensuring that important users (in industry, community life and public administration) are incorporated into/take part in dissemination activities for the project.

When assessing dissemination and communication plans, importance should be attached to the level of detail provided and how realistic the plans are.

The dissemination plans are very good.

Selected mark : A - Very good
The project’s dissemination and communication plans provide a thorough level of detail and are of high relevance.

Overall assessment of the referee/panel

How does the project rank in terms of the referee’s/panel’s overall assessment?

This criterion indicates the overall view of the referee/panel, based on the specific criteria which they have been asked to assess.

This is a very well described project that draws together an international team with appropriate expertise and track records. The questions being tackled are important and will help improve understanding of physical activity in childhood cancer survivors – and whether this offers a potential route to help improve health outcomes in this population. The work that is described is a logical first step and will lead to important publications. Some of the stated benefits will not be realised through this project (alone) and will require subsequent work. This does not diminish the importance of the proposed research as it represents a logical first step towards longer term benefit and impact.

Selected mark : 6 - Excellent
A project at a very high international level and of great national and international interest. Publications in leading journals are expected. The researchers are among the leaders in their field.

Special points to consider

Comments to special points to consider
